# Semaphorin 3A mitigates lipopolysaccharide-induced chondrocyte inflammation, apoptosis and extracellular matrix degradation by binding to Neuropilin-1

**DOI:** 10.1080/21655979.2021.1974806

**Published:** 2021-11-25

**Authors:** Huiyu Zhang, Yue Lu, BingBing Wu, Fei Xia

**Affiliations:** a Department of Hand Surgery, Affiliated Hospital 2 of Nantong University, Nantong, Jiangsu, China; b Department of Orthopedics, Affiliated Hospital of Nantong University, Nantong, Jiangsu, China

**Keywords:** SEMA3A, nrp-1, chondrocyte apoptosis, extracellular matrix degradation, osteoarthritis

## Abstract

Semaphorin 3A (SEMA3A) and its receptor neuropilin-1 (NRP-1) are expressed low in chondrocytes under stress, and overexpressing SEMA3A reduces pro-inflammatory cytokine release. This study was aimed at exploring whether SEMA3A participates in lipopolysaccharide (LPS)-induced chondrocyte inflammation, apoptosis and extracellular matrix (ECM) degradation. SEMA3A and NRP-1 expression in LPS-induced ATDC5 cells was determined with RT-qPCR and western blotting. Following stimulation with LPS in the absence or presence of SEMA3A overexpression, the viability of ATDC5 cells was observed through CCK-8 assay. RT-qPCR and western blot were performed to detect the expression of pro-inflammatory cytokines. ATDC5 cell apoptosis was observed through TUNEL, and apoptosis-related proteins were assayed. Expression of ECM-related proteins was measured by RT-qPCR and western blotting. Additionally, the binding of SEMA3A to NRP-1 was verified by co-immunoprecipitation. After interference with NRP-1, cell viability, inflammation and ECM degradation were examined in LPS-induced ATDC5 cells with SEMA3A overexpression. Results revealed that SEMA3A expression in ATDC5 cells decreased following stimulation with LPS. Overexpressing SEMA3A improved cell viability and reduced the inflammatory injury of LPS-stimulated ATDC5 cells. Moreover, SEMA3A overexpression alleviated LPS-induced apoptosis and ECM degradation of ATDC5 chondrocytes. SEMA3A and NRP-1 bound to each other in ATDC5 cells. NRP-1 interference crippled the ameliorative effect of SEMA3A overexpression on LPS-induced chondrocyte inflammation, apoptosis and ECM degradation. To conclude, SEMA3A binds to NRP-1, mitigating LPS-induced chondrocyte inflammation, apoptosis and ECM degradation. This study elucidated the role of SEMA3A in osteoarthritis and illustrated its action mechanism involving NRP-1.

## Introduction

Osteoarthritis (OA) is a chronic joint disease specifically on the site of cartilage, clinically presented as joint pain, stiffness and hypertrophy and limited joint mobility [1]. As OA slowly progresses, it will eventually affect the joint structure, resulting in cartilage destruction, fibrosis, fracture and damage to the entire articular surface [2]. It is believed that OA is the result of the combination of multiple risk factors including genetics, age, obesity and mechanical trauma [3,4]. The onset of OA is mostly seen in the middle-aged and the elderly and is a leading cause of disability in the elderly [5]. China, along with many other countries, is now facing the reality of aging society and growing public healthcare burden relevant to aging population [6,7]. According to an analysis, the incidence of symptomatic knee OA is estimated to be 8.1% in Chinese population with a mean age of 59.8 years [8]. As the national population ages, this number is expected to rise continuously in the next decade. Other than the treatment options aiming for symptom alleviation and surgical replacement of the joint, there is so far no pharmacological means of cartilage repair or injury reversion [9,10]. Therefore, it is urgent to identify key genes in OA that have therapeutic values for a precise and effective treatment.

Currently known pathological features of osteoarthritis are mainly degeneration of articular cartilage, reduction of cartilage extracellular matrix and long-term low-grade inflammation [11–13]. Chondrocytes are the dominant cell type in cartilage tissue, which participate in the integrity of cartilage function and maintain the balance between cartilage injury and remodeling [14]. Type II collagen (collagen II) is one of the primary extracellular matrix degradation (ECM) macromol-ecules in cartilage. Matrix metalloproteinases (MMPs) are a class of enzymes with a specific function that can hydrolyze the triple helix structure in type II collagen and maintain the balance between synthesis and degradation in normal cartilage ECM [15]. Previous works have noted the upregulation of MMPs (MMP3, MMP9 and MMP13) and aggrecanase a disintegrin and metalloproteinase with thrombospondin motifs (ADAMTS) in the chondrocytes of OA patients, which are important reference indexes for assessing the state of cartilage matrix [16–18]. Additionally, the apoptosis of chondrocytes contributes to cartilage degradation, thus playing an essential role in the pathogenesis of OA [19]. Inhibition or reversal of these pathological processes is considered to be an effective means of OA treatment.

Previous study has revealed a time-dependent decrease in the expression of Semaphorin 3A (SEMA3A) and Neuropilin-1 (NRP-1) in chondrocytes under mechanical stress [20]. The same study has also described the suppressive effect of SEMA3A overexpression on stress-induced release of pro-inflammatory cytokines. SEMA3A is known to be an osteoprotective factor that helps to maintain the balanced function of osteoblasts and osteoclasts [21]. Immunoregulatory NRP-1 is a receptor of SEMA3A that plays a simulative role in osteogenesis [22,23]. The interaction between SEMA3A and NRP-1 has been reported in a series of diseases, such as osteoporosis [24], leukemia [25], colorectal carcinoma and so on [26]. However, the function of their interaction remains to be explored in chondrocytes challenged with lipopolysaccharide (LPS), a major inflammasome associated with the progression of OA [27].

This study was designed to elucidate the roles of SEMA3A and NRP-1 in LPS-induced *in vitro* model of OA and to provide potential therapeutic targets for OA treatment.

## Materials and methods

### Cell culture and treatment

ATDC5 mouse chondrocytes (SNL-178; Sunncell, Wuhan, China) were cultured in the complete culture medium containing Dulbecco’s modified Eagle’s medium (DMEM)/F12 (Gibco, Carlsbad, CA, USA) supplemented with 10% fetal bovine serum (FBS; Gibco, NY, USA) and 1% penicillin-streptomycin liquid in an environment of 95% air and 5% CO_2_. LPS (Solarbio, Beijing, China) in the concentration of 5 μg/mL was chosen to stimulate ATDC5 cells for 6, 12 and 24 h before observation, as previously described [28].

### Cell transfection

SEMA3A pcDNA 3.1 plasmid (Ov-SEMA3A), empty vector pcDNA3.1 plasmid (Ov-NC), two small interfering RNAs (siRNA) targeting NRP-1 (siRNA-NRP-1-1 and siRNA-NRP-1-2) and the negative control (siRNA-NC) were obtained from GenePharma (Shanghai, China). Lipofectamine 2000 reagent (Invitrogen, Carlsbad, CA, USA) was used for cell transfection in accordance with the manufacturer’s protocol. Successfully transfection was evaluated with reverse transcription-quantitative polymerase chain reaction (RT-qPCR) and western blot analysis.

### Cell counting kit 8 (CCK-8) assay

CCK-8 Cell Proliferation and Cytotoxicity Assay Kit (CA1210; Solarbio) was used for the test of chondrocyte viability. Cell suspension was added into a 96-well plate (100 µL/well) and incubated in an environment of 95% air and 5% CO_2_. After addition of CCK-8 solution (10 µL/well), the incubation was continued for 2 h. A microplate reader was used to measure the absorbance at 450 nm.

### TdT-mediated dUTP Nick-End Labeling (TUNEL) staining

The TUNEL assay kit (Beyotime, Shanghai, China) was used to detect apoptotic ATDC5 cells, in accordance with the specification provided by the supplier. Briefly, Cells were fixed by 4% paraformaldehyde on a coverslip cells, followed by incubation with 50 μL TUNEL reaction buffer for 1 h at 37°C. The nuclei were counterstained with DAPI in the dark. After the coverslip was blocked by anti-fade solution, the photos of TUNEL-positive cells (green fluorescence) were captured by fluorescence microscopy (Olympus Corporation) and counted using by Image J software (Bio-Rad, Hercules, CA, USA).

### Co-Immunoprecipitation (Co-IP) analysis

The binding relationship between SEMA3A and NRP-1 was examined through IP assay. The antibody was mixed with cell lysate containing the target protein and incubated at room temperature for 60 min. Protein A/G Agarose Resin (Yeasen, Shanghai, China) was added into a 2 mL tube and centrifuged at 500 rpm for 1 min, and the supernatant was discarded. Following resuspension, the antigen-antibody mixture was added to the resin, sufficiently mixed and centrifuged to collect the supernatant. After elution of the sample, the precipitate is detected.

### RT-qPCR assay

Total RNA was extracted from ATDC5 cells using RIzol reagent (Invitrogen; Thermo Fisher Scientific, Inc.) following the instructions. Reverse transcription was then performed utilizing PrimeScript™ RT reagent Kit with gDNA Eraser (TaKaRa, Tokyo, Japan) to synthesize complementary DNA (cDNA). PCR was performed using SYBR Green PCR Master Mix (Applied Biosystems) on an ABI 7300 thermal-recycler (Applied Biosystems; Thermo Fisher Scientific, Inc.). GAPDH was used as internal controls for normalization. The relative mRNA expression was calculated using the 2^−ΔΔCq^ method [29].

### Western blot analysis

Cell lysate was prepared on ice by the use of lysis buffer RIPA lysis buffer (Beyotime, Shanghai, China) followed by centrifugation. After analysis of protein concentration using a bicinchoninic acid kit (Beyotime, Shanghai, China), an equal amount of protein in each group was loaded on 10% sodium dodecyl sulfate (SDS)-polyacrylamide gels, followed by electrophorized and transferred to a polyvinylidene fluoride membranes (PVDF; EMD Millipore, Bedford, MA, USA). Following blockade by 5% nonfat milk, the membrane was incubated with respective diluted primary antibodies and secondary antibody. The proteins were finally visualized under enhanced chemiluminescence (EMD Millipore). Parallel blotting of GAPDH was used as the internal control.

### Statistical analysis

Statistical analysis was conducted on GraphPad Prism 8.0, and data were presented as mean ± standard deviation (SD). Comparisons between two groups were evaluated using student’s t-test. Differences among the results of multiple groups were evaluated by one-way one-away analysis of variance (ANOVA) followed by Tukey’s post hoc test. P < 0.05 represents significant difference.

## Results

### SEMA3A and NRP-1 expression decreased in ATDC5 chondrocytes under LPS stimulation

**Figure 1.**
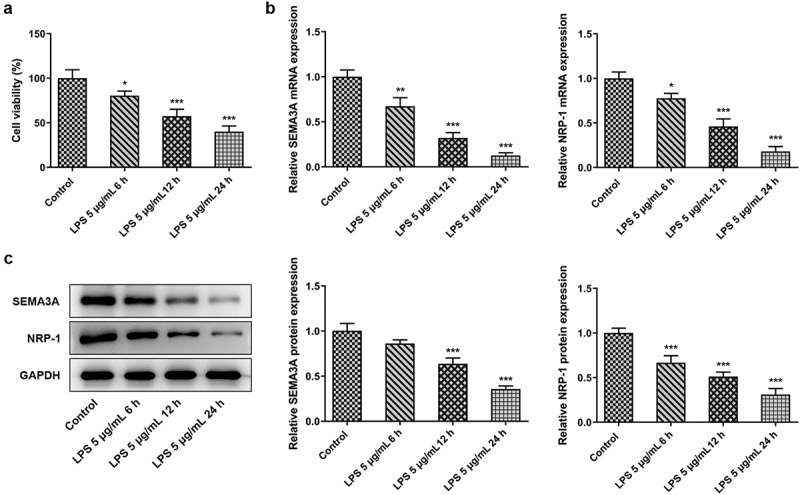
SEMA3A expression decreased in ATDC5 cells under LPS stimulation. (a) Cell viability was observed via CCK-8 assay following stimulation with LPS (5 ug/mL) for 6, 12 and 24 h. (b-c) SEMA3A and NRP-1 expression was detected by RT-qPCR and western blot following stimulation with LPS (5 ug/mL) for 6, 12 and 24 h. *P < 0.05, **P < 0.01, ***P < 0.001 vs. control

SEMA3A is known to be an osteoprotective factor that helps to maintain the balanced function of osteoblasts and osteoclasts [21]. NRP-1 is a receptor of SEMA3A that plays a simulative role in osteogenesis [22,23]. The interaction between SEMA3A and NRP-1 has been reported in primary rat calvarial osteoblast induced by high-dose dexamethasone [24]. However, the function of their interaction remains to be explored in LPS-induced chondrocytes. Firstly, cell viability was evaluated by CCK-8 assay after ATDC5 cells exposed to LPS for 6, 12 and 24 h. It was first observed that the viability of ATDC5 cells was notably decreased by LPS challenge in a time-dependent manner compared with the control group (Figure 1a). Moreover, the expression of SEMA3A and NRP-1 was tested using RT-qPCR and western blot assays in the presence of LPS. As displayed in Figure 1b-C, LPS induction led to remarkably downregulated SEMA3A and NRP-1 mRNA and protein expression in ATDC5 cells in a time-dependent manner. These results imply the important roles of SEMA3A and NRP-1 in LPS-stimulated ATDC5 chondrocytes.

**SEMA3A overexpression ameliorated LPS-induced viability injury and inflammation of ATDC5 chondrocytes**

**Figure 2.**
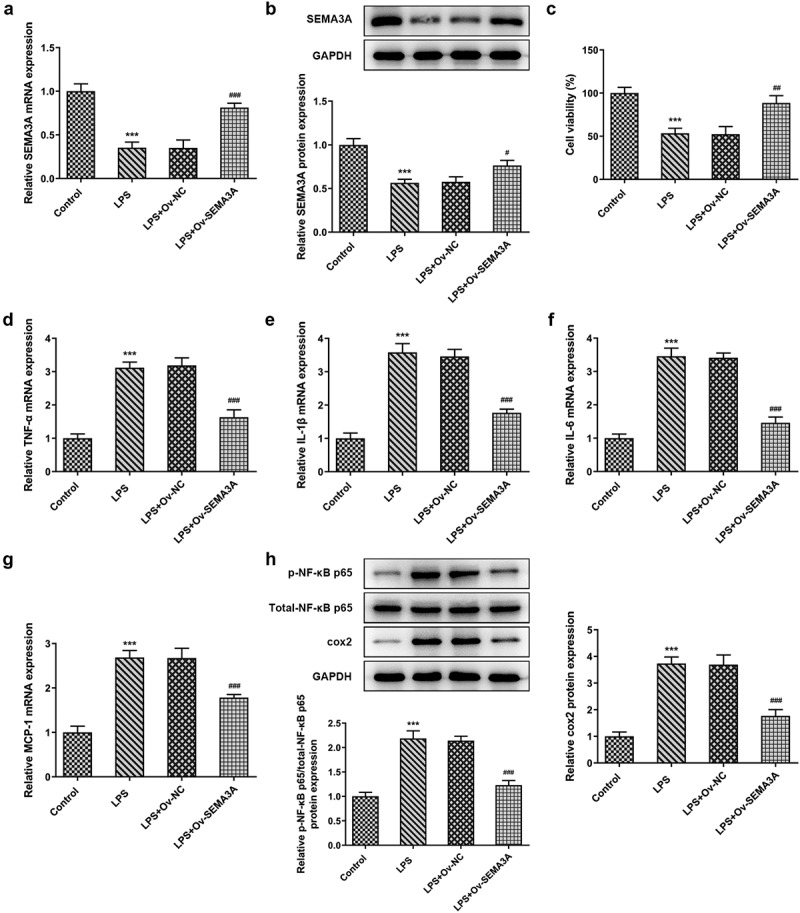
SEMA3A overexpression improved cell viability and alleviated inflammatory injury of ATDC5 cells stimulated with LPS (5 ug/mL) for 12 h. (a-b) SEMA3A expression was detected by RT-qPCR and western blot after transfection of pcDNA 3.1(+)/SEMA3A into LPS-stimulated ATDC5 cells. (c) Cell viability was detected by the use of CCK-8. (d-g) The release of pro-inflammatory cytokines was assayed by RT-qPCR. (h) The expression of inflammatory mediator p-NF-κB p65, NF-κB p65 and COX2 was determined by western blot analysis. ***P < 0.001 vs. control; ^#^P < 0.05, ^##^P < 0.01, ^###^P < 0.001 vs. LPS+Ov-NC

To explore the effects of SEMA3A on LPS-induced viability injury and inflammation of ATDC5 chondrocytes, SEMA3A was overexpressed by transfection with SEMA3A plasmid. After transfection of SEMA3A overexpressing plasmid into ATDC5 cells, notably increased SEMA3A expression was determined by both RT-qPCR and western blot (Figure 2a-b). The result of CCK-8 assay showed that while LPS stimulation led to the loss of viability of ATDC5 cells as comparison to control group, overexpressing SEMA3A was able to effectively protect ATDC5 cells from this impact (Figure 2c). Meanwhile, the release of pro-inflammatory cytokines tumor necrosis factor-α (TNF-α), interleukin (IL)-1β, IL-6 and monocyte chemoattractant protein-1 (MCP-1) was found to be greatly promoted by LPS compared to the control group, whereas SEMA3A overexpression prominently inhibited their release in ATDC5 cells (Figure 2d-g). The same effect of SEMA3A was also observed on the protein expression of inflammatory mediator phospho (p)-nuclear factor (NF)-κB p65 and cyclooxygenase2 (COX2) (Figure 2h). Thus, SEMA3A overexpression ameliorated LPS-induced viability injury and inflammation of ATDC5 cells.

### SEMA3A overexpression suppressed LPS-induced chondrocyte apoptosis and cartilage matrix degradation

**Figure 3.**
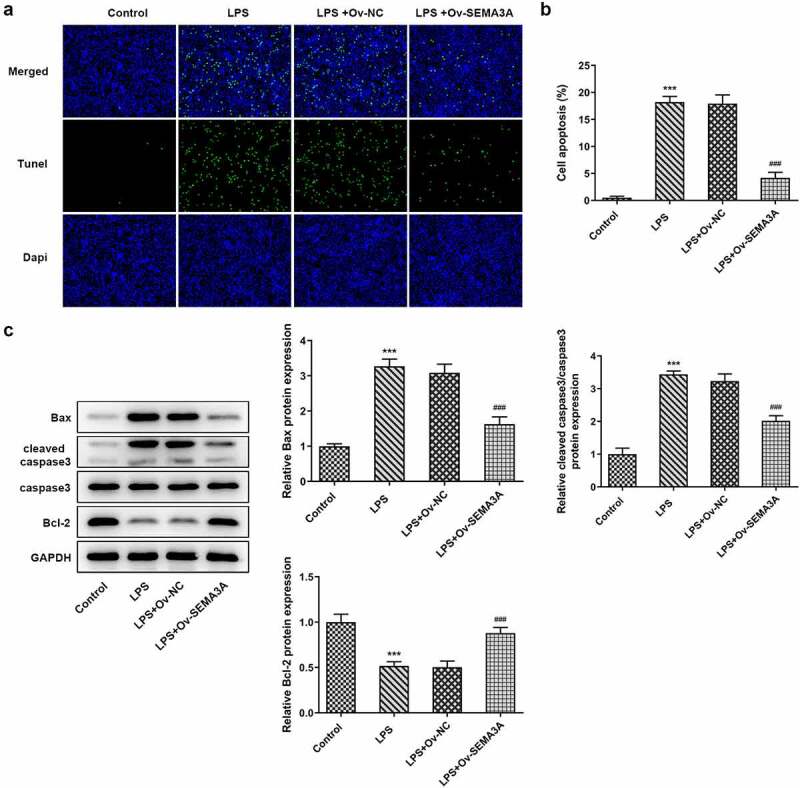
SEMA3A overexpression alleviated LPS-induced ATDC5 cell apoptosis. (a-b) The apoptosis of LPS-stimulated ATDC5 cells was observed though TUNEL assay after overexpression of SEMA3A. (c) The protein expression of pro-apoptotic Bax and cleaved caspase3 and that of anti-apoptotic Bcl-2 was detected by western blotting. ***P < 0.001 vs. control; ^###^P < 0.001 vs. LPS+Ov-NC

**Figure 4.**
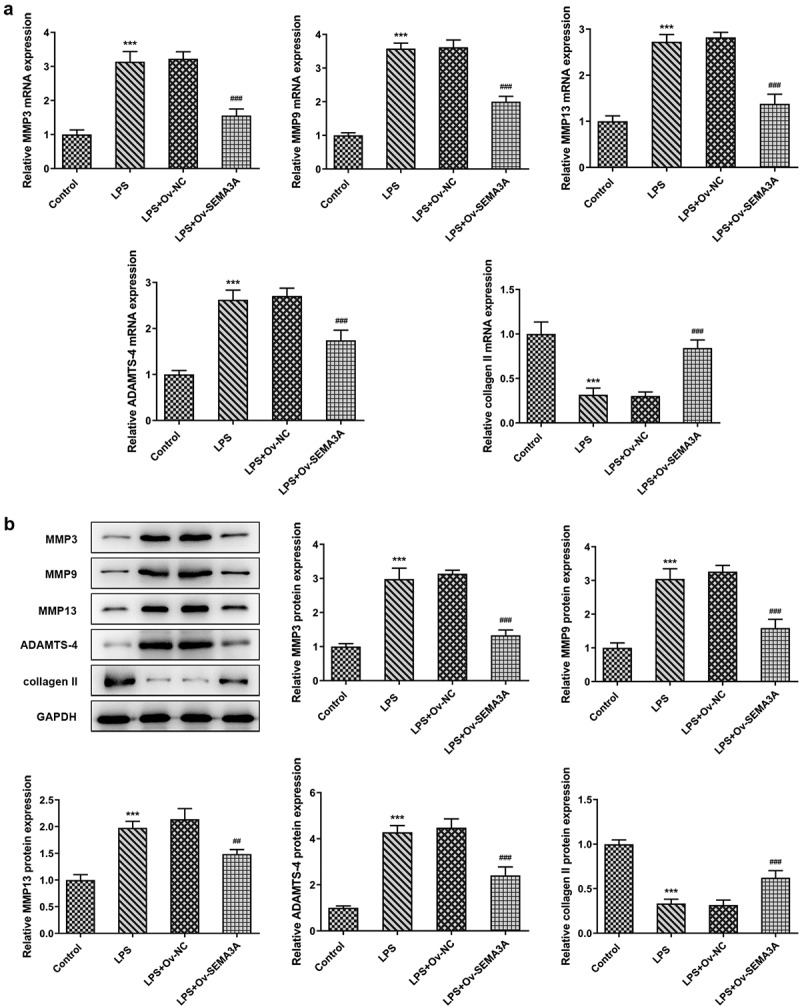
SEMA3A overexpression attenuated LPS-induced ATDC5 cell cartilage matrix degradation. (a-b) ECM-degrading MMP3, MMP9, MMP13 and ADAMTS-4 as well as collagen II, the main component of ECM, were assayed by RT-qPCR and western blot. ***P < 0.001 vs. control; ^##^P < 0.01, ^###^P < 0.001 vs. LPS+Ov-NC

Subsequently, the effects of SEMA3A overexpression on LPS-induced chondrocyte apoptosis and cartilage matrix degradation were evaluated. Following LPS stimulation, the number of TUNEL positive ATDC5 cells increased significantly when compared to the control group, which however showed a noticeable decline in the presence of SEMA3A overexpression (Figure 3a-b). Additionally, western blot assay detected an elevation in the expression of Bcl-2-associated X (Bax) and cleaved caspase3 and a reduction in that of B-cell lymphoma-2 (Bcl-2) in LPS-stimulated ATDC5 cells compared to the control group, as well as an opposite trend of their expression after overexpression of SEMA3A (Figure 3c). Furthermore, in LPS-stimulated ATDC5 chondrocytes, the expression of ECM protein-degrading matrix metalloproteinases (MMP3, MMP9, MMP13 and ADAMTS-4) was abnormally high while that of collagen II, the principal molecular component of ECM, was extremely low relative to the control group (Figure 4a-b). However, SEMA3A overexpression inhibited the expression of MMP3, MMP9, MMP13 and ADAMTS-4 and enhanced that of collagen II in LPS-stimulated ATDC5 chondrocytes when compared to the LPS+Ov-NC group. These results suggest that SEMA3A overexpression suppressed LPS-induced chondrocyte apoptosis and cartilage matrix degradation.

### SEMA3A and NRP-1 bound in ATDC5 chondrocytes

**Figure 5.**
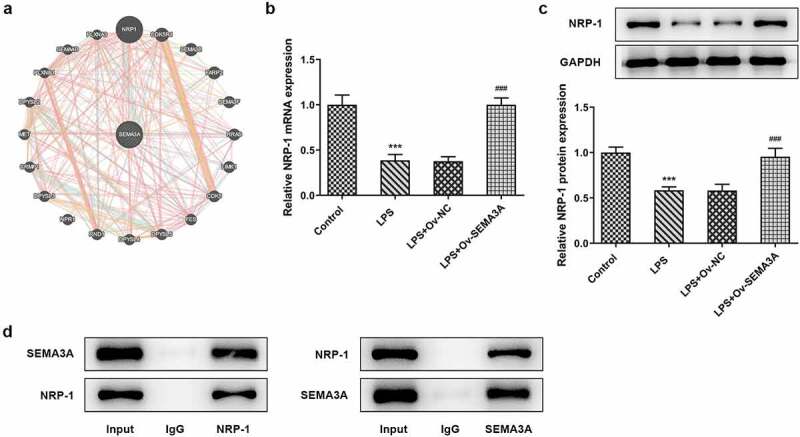
SEMA3A and NRP-1 bound to each other in ATDC5 cells. (a) Predicted binding of SEMA3A to NRP-1 on GeneMANIA database. (b-c) NRP-1 expression was detected by RT-qPCR and western blot assay in LPS-stimulated ATDC5 cells in the absence or presence of SEMA3A overexpression. ***P < 0.001 vs. control; ^###^P < 0.001 vs. LPS+Ov-NC. (d) The binding relationship between SEMA3A and NRP-1 was verified by co-IP assay

The binding relationship between SEMA3A and NRP-1 (Figure 5a) was predicted by GeneMANIA database (http://genemania.org/). Both the mRNA and protein expression of NRP-1 were found to be downregulated by LPS stimulation but notably upregulated by SEMA3A overexpression in ATDC5 cells (Figure 5b-c). The protein interactome of anti-NRP-1 and SEMA3A as well as that of anti-SEMA3A and NRP-1 were confirmed by the result of co-IP assay (Figure 5d). The binding of SEMA3A and NRP-1 was thus demonstrated in ATDC5 chondrocytes.

**NRP-1 interference diminished the ameliorative effect of SEMA3A overexpression on LPS-induced chondrocyte viability injury and inflammation**

**Figure 6.**
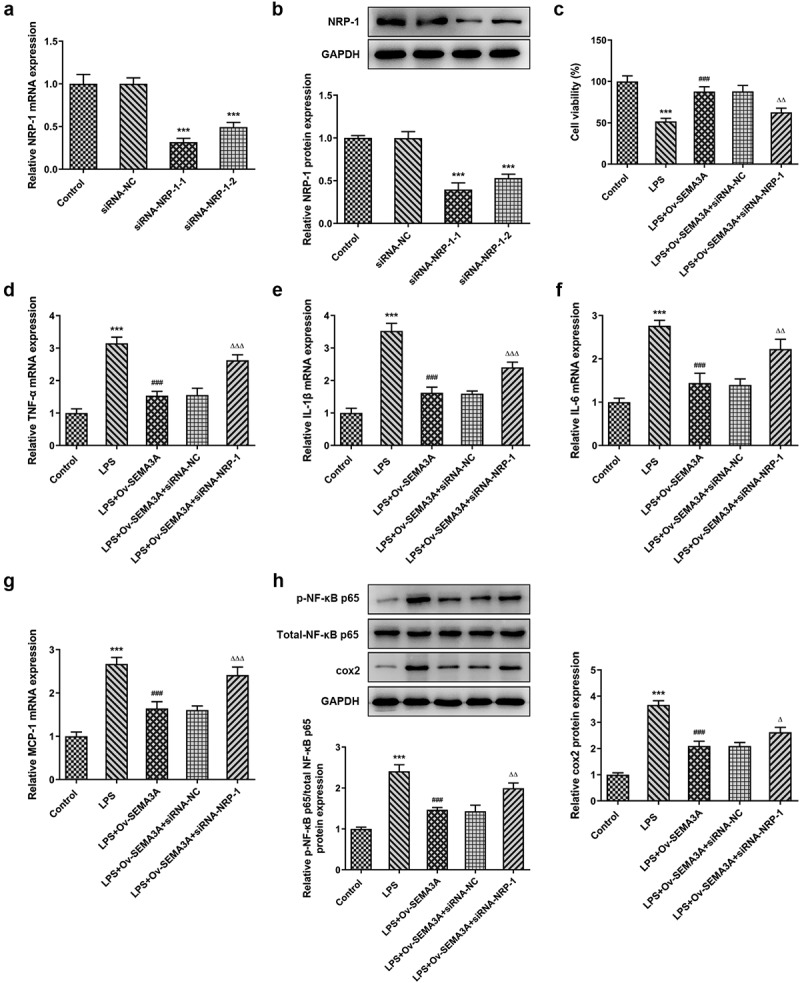
NRP-1 interference reversed SEMA3A-ameliorated ATDC5 viability injury and inflammation induced by LPS. (a-b) NRP-1 interference level was detected by RT-qPCR and western blot after transfection of siRNAs targeting NRP-1 (siRNA-NRP-1-1/2). ***P < 0.001 vs. siRNA-NC. (c) The viability of LPS-stimulated ATDC5 cells following SEMA3A overexpression with or without NRP-1 knockdown was detected by CCK-8 assay. (d-g) The expression of pro-inflammatory cytokines was detected by RT-qPCR and western blot. (h) The expression of inflammatory mediator p-NF-κB p65, NF-κB p65 and COX2 was determined by western blot analysis. ***P < 0.001 vs. control; ^###^P < 0.001 vs. LPS; ^Δ^P<0.05, ^ΔΔ^P<0.01, ^ΔΔΔ^P<0.001 vs. LPS+Ov-SEMA3A+siRNA-NC

To further investigate the function of NRP-1 in its interaction with SEMA3A, siRNA-NRP-1-1 and siRNA-NRP-1-2 were constructed and respectively transfected into ATDC5 cells. RT-qPCR and western blot confirmed the interference efficacy and a better knockdown effect of siRNA-NRP-1-1 (Figure 6a-b), which was selected for the subsequent experiments. Following transfection of siRNA-NRP-1, the viability of LPS-stimulated ATDC5 cells noticeably decreased, by contrasted with the LPS-Ov-SEMA3A+siRNA-NC group (Figure 6c). Meanwhile, SEMA3A overexpression-downregulated pro-inflammatory cytokine (TNF-α, IL-1β, IL-6 and MCP-1) and inflammatory mediators (p-NF-κB p65 and COX) expression was also largely upregulated by NRP-1 knockdown in LPS-stimulated ATDC5 chondrocytes (Figure 6d-h). These results suggest that NRP-1 interference diminished the ameliorative effect of SEMA3A overexpression on LPS-induced chondrocyte viability injury and inflammation.

**NRP-1 interference diminished the ameliorative effect of SEMA3A overexpression on LPS-induced chondrocyte apoptosis and cartilage matrix degradation**

**Figure 7.**
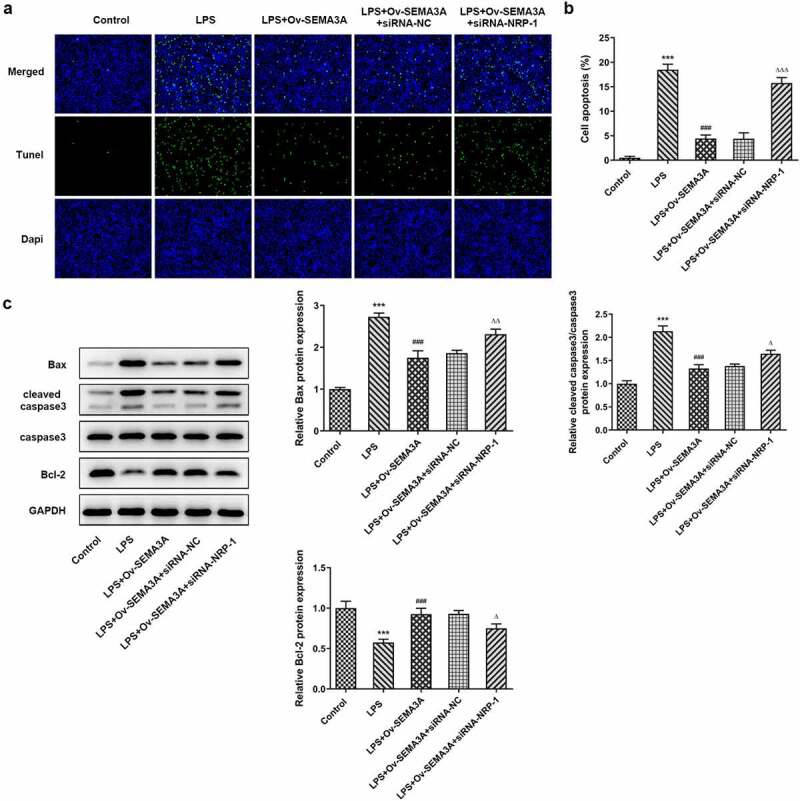
NRP-1 interference reversed SEMA3A-ameliorated ATDC5 apoptosis induced by LPS. (a-b) The apoptosis of LPS-stimulated ATDC5 cells following SEMA3A overexpression with or without NRP-1 knockdown was observed via TUNEL assay. (c) Apoptosis-related protein expression was determined by western blot analysis. ***P < 0.001 vs. control; ^###^P < 0.001 vs. LPS; ^Δ^P<0.05, ^ΔΔ^P<0.01, ^ΔΔΔ^P<0.001 vs. LPS+Ov-SEMA3A+siRNA-NC

**Figure 8.**
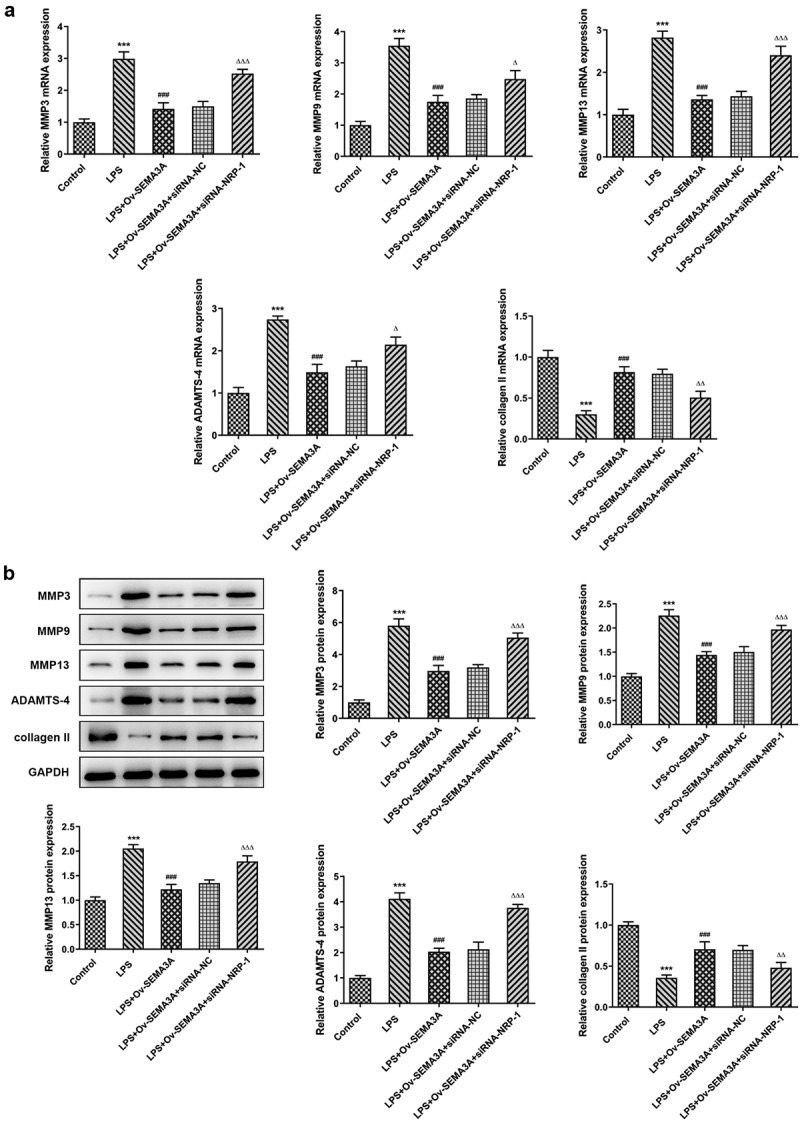
NRP-1 interference reversed SEMA3A-ameliorated ATDC5 cartilage matrix degradation induced by LPS. (a-b) The expression of MMP3, MMP9, MMP13, ADAMTS-4 and collagen II was detected by RT-qPCR and western blot to reflect the state of extracellular matrix degradation. ***P < 0.001 vs. control; ^###^P < 0.001 vs. LPS; ^Δ^P<0.05, ^ΔΔ^P<0.01, ^ΔΔΔ^P<0.001 vs. LPS+Ov-SEMA3A+siRNA-NC

The further experiments investigated the regulatory effects of SEMA3A and NRP-1 in LPS-induced chondrocyte apoptosis and cartilage matrix degradation. In comparison with LPS-induced ATDC5 cells transfected solely with ov-SEMA3A, those transfected with both Ov-SEMA3A and siRNA-NPR-1 exhibited much higher apoptosis level (Figure 7a-b). Consistently, the expression of apoptosis markers Bax and cleaved caspase3 was notably higher in the siRNA-NPR-1 group than that in the siRNA-NC group, whereas the expression of anti-apoptotic Bcl-2 showed the opposite (Figure 7c). Moreover, although SEMA3A overexpression decreased the expression of MMP3, MMP9, MMP13 and ADAMTS-4 and increased collagen II expression in LPS-stimulated ATDC5 cells, these changes were offset in the presence of NRP-1 knockdown (Figure 8a-b). These results indicate that NRP-1 interference diminished the ameliorative effect of SEMA3A overexpression on LPS-induced chondrocyte apoptosis and cartilage matrix degradation.

## Discussion

Osteoarthritis (OA) is an age-related bone disease frequently seen in clinical cases. Chronic joint pain in OA, the major cause of loss of mobility in the elderly, is connected with not only the ongoing joint deformity but also the fluctuating degree of local inflammation [30]. The present study demonstrated that SEMA3A could bind to NRP-1, mitigating LPS-induced chondrocyte inflammation, apoptosis and ECM degradation.

A recent study has found that the expression of SEMA3A and its receptor NRP-1 decreased in a time-dependent manner in mechanical stress-stimulated chondrocytes, and that overexpressing SEMA3A could reduce mechanical stress-induced release of inflammatory cytokines in those chondrocytes [20]. Moreover, a decline in the expression of SEMA3A/NRP-1 signaling has been reported earlier in rats with periapical lesions as well as patients with apical periodontitis [31]. Our preliminary investigation found that SEMA3A also had a meaningful expression in LPS-challenged chondrocytes, as its expression decreased with the increase in the duration of LPS stimulation. Additionally, it has been reported multiple times that SEMA3A plays an ameliorating role against inflammatory response. For example, Rienks et al. found through a murine model of myocardial infarction that SEMA3A improve the inflammatory state of mouse bone marrow-derived macrophages [32]. Huang et al. reported that in radiation-induced osteoporosis SEMA3A suppresses inflammation and prevents the differentiation of macrophages into osteoclasts [33]. Similarly, it was demonstrated in our study that overexpression of SEMA3A not only increased the viability of LPS-stimulated chondrocytes but significantly downregulated the level of pro-inflammatory cytokines. Therefore, SEMA3A likely plays a protective role against the inflammatory injury of chondrocytes in OA.

Studies have also substantiated the role of SEMA3A as a regulator of cell apoptosis in both cancers and non-cancerous diseases. In head and neck squamous cell carcinoma (HNSCC), SEMA3A functions to suppress tumor growth partly by accelerating HNSCC apoptosis [34]. On the other hand, SEMA3A has been shown to promote the apoptosis of fibroblast-like synoviocytes and thus obstructs osteoclastogenesis in rheumatoid arthritis [35]. Another study has determined that aberrantly high SEMA3A overexpression could be related to increased chondrocyte apoptosis in knee OA [36]. Opposite to its pro-apoptotic effect described in the above studies, SEMA3A overexpression in our study effectively suppressed LPS-induced apoptosis of chondrocytes while downregulating the expression of pro-apoptotic proteins and upregulating that of anti-apoptotic Bcl-2.

Previous research has revealed that SEMA3A interacting with Schwann cells facilitates extracellular matrix mineralization, thereby contributing to the osteogenesis of MG63 cells [37]. The expression of MMP3, MMP9, MMP13 and ADAMTS-4, participants in the degradation of extracellular matrix, is an important reference index for assessing the state of cartilage matrix [16]. A study of OA has illustrated the downregulation of MMP1 and MMP3 in OA cartilage tissue-derived chondrocytes in the presence of additional SEMA3A [38]. Consistent with previous studies, our results showed downregulated expression of MMP3, MMP9, MMP13 and ADAMTS-4 but upregulated collagen II in LPS-stimulated chondrocytes after overexpression of SEMA3A.

To further explore the underlying mechanism of SEMA3A alleviating LPS-induced chondrocyte inflammation and apoptosis, GeneMANIA database was consulted for its potential interaction network. The result showed that SEMA3A could bind to its receptor NRP-1, which was confirmed in the present study. According to earlier research, NRP1 is likely to play a dual role in the process of osteoblasts differentiating into osteocytes, directly promoting differentiation while maintains the fine balance in osteogenesis [39]. Furthermore, in a mouse model of hepatotoxicity, upregulation of SEMA3A and its receptor NRP-1 synergistically inhibited the Akt2 /NF-κB and NLRP3 inflammatory signaling pathways [40]. This positive correlation between SEMA3A and NRP-1 was also observed in the present study, in addition to the diminished effect of SEMA3A overexpression by NRP-1 depletion on LPS-induced chondrocyte inflammation and apoptosis as well as cartilage matrix degradation.

## Conclusion

In brief, SEMA3A bound to its receptor NRP-1 in chondrocytes, alleviating LPS-induced inflammation, apoptosis and extracellular matrix degradation. This finding complements previous studies, sheds light on the mechanism of SEMA3A-NRP-1 interaction in OA and provides possible targets for OA treatment.

## References

[1] Ordeberg G. Characterization of joint pain in human OA. Novartis Found Symp. 2004;260:105–115. discussion 115-121, 277-109. 15283446

[2] Shelton LR. A closer look at osteoarthritis. Nurse Pract. 2013;38(7):30–36. quiz 36-37. 10.1097/01.NPR.0000431178.49311.42 23728492

[3] Ye ZZ, Zhang ZY, Li ZG, et al. Toward wiping out osteoarthritis in China: research highlights. Chin Med J (Engl). 2020;133(8):883–885. 32187051 10.1097/CM9.0000000000000746 PMC7176452

[4] Swift A. Osteoarthritis 1: Physiology, risk factors and causes of pain. Nurs Times. 2012;108:12–15. 22479933

[5] Sun X, Zhen X, Hu X, et al. Osteoarthritis in the middle-aged and elderly in china: Prevalence and influencing factors. Int J Environ Res Public Health. 2019;16. 10.3390/ijerph16234701 PMC6926632 31779104

[6] Fang EF, Scheibye-Knudsen M, Jahn HJ, et al. A research agenda for aging in China in the 21st century. Ageing Res Rev. 2015;24:197–205. 26304837 10.1016/j.arr.2015.08.003 PMC5179143

[7] Chen R, Xu P, Li F, et al. Internal migration and regional differences of population aging: An empirical study of 287 cities in China. Biosci Trends. 2018;12(2):132–141. 29607873 10.5582/bst.2017.01246

[8] Tang X, Wang S, Zhan S, et al. The prevalence of symptomatic knee osteoarthritis in china: Results from the china health and retirement longitudinal study. Arthritis Rheumatol. 2016;68(3):648–653. 26474054 10.1002/art.39465

[9] Taruc-Uy RL, Lynch SA. Diagnosis and treatment of osteoarthritis. Prim Care. 2013;40(4):821–836. vii. 24209720 10.1016/j.pop.2013.08.003

[10] Rychel JK. Diagnosis and treatment of osteoarthritis. Top Companion Anim Med. 2010;25(1):20–25. 20188335 10.1053/j.tcam.2009.10.005

[11] Robinson WH, Lepus CM, Wang Q, et al. Low-grade inflammation as a key mediator of the pathogenesis of osteoarthritis. Nat Rev Rheumatol. 2016;12(10):580–592. 27539668 10.1038/nrrheum.2016.136 PMC5500215

[12] Pearle AD. Warren RF and Rodeo SA. Basic science of articular cartilage and osteoarthritis. Clin Sports Med. 2005;24(1):1–12. 15636773 10.1016/j.csm.2004.08.007

[13] Guilak F, Nims RJ, Dicks A. Wu CL and Meulenbelt I. Osteoarthritis as a disease of the cartilage pericellular matrix. Matrix Biol. 2018;71-72:40–50. 29800616 10.1016/j.matbio.2018.05.008 PMC6146061

[14] Jiang Y, Tuan RS. Origin and function of cartilage stem/progenitor cells in osteoarthritis. Nat Rev Rheumatol. 2015;11(4):206–212. 25536487 10.1038/nrrheum.2014.200 PMC5413931

[15] Liu S, Yang H, Hu B, et al. Sirt1 regulates apoptosis and extracellular matrix degradation in resveratrol-treated osteoarthritis chondrocytes via the Wnt/β-catenin signaling pathways. Exp Ther Med. 2017;14:5057–5062. 29201214 10.3892/etm.2017.5165 PMC5704318

[16] Takaishi H, Kimura T, Dalal S, et al. Joint diseases and matrix metalloproteinases: a role for MMP-13. Curr Pharm Biotechnol. 2008;9(1):47–54. 18289056 10.2174/138920108783497659

[17] Wang DW, Lin N, Tang YC, et al. Inhibition of P2Y11R ameliorated TNF-alpha-induced degradation of extracellular matrix in human chondrocytic SW1353 cells. Am J Transl Res. 2019;11:2108–2116. 31105822 PMC6511765

[18] Attur M, Yang Q, Shimada K, et al. Elevated expression of periostin in human osteoarthritic cartilage and its potential role in matrix degradation via matrix metalloproteinase-13. Faseb J. 2015;29(10):4107–4121. 26092928 10.1096/fj.15-272427 PMC4566939

[19] Hwang HS, Kim HA. Chondrocyte apoptosis in the pathogenesis of osteoarthritis. Int J Mol Sci. 2015;16(11):26035–26054. 26528972 10.3390/ijms161125943 PMC4661802

[20] Sumi C, Hirose N, Yanoshita M, et al. Semaphorin 3A inhibits inflammation in chondrocytes under excessive mechanical stress. Mediators Inflamm. 2018;5703651:2018. 10.1155/2018/5703651 PMC5911320 29849491

[21] Hayashi M, Nakashima T, Taniguchi M, Kumanogoh A and Takayanagi H, et al. Osteoprotection by semaphorin 3A. Nature. 2012;485(7396):69–74. 22522930 10.1038/nature11000

[22] Romeo PH. Lemarchandel V and Tordjman R. Neuropilin-1 in the immune system. Adv Exp Med Biol. 2002;515:49–54. 12613542 10.1007/978-1-4615-0119-0_4

[23] Song Y, Liu X, Feng X, et al. NRP1 accelerates odontoblast differentiation of dental pulp stem cells through classical wnt/beta-catenin signaling. Cell Reprogram. 2017;19(5):324–330. 28910136 10.1089/cell.2017.0020

[24] Zheng L. Luteolin stimulates proliferation and inhibits late differentiation of primary rat calvarial osteoblast induced by high-dose dexamethasone via sema3A /NRP1/Pleixin A1. Curr Pharm Biotechnol. 2020;22:1538-1545. 10.2174/1389201021666201216150442 33327910

[25] Palodetto B, da Silva Santos Duarte A, Rodrigues LM, et al. SEMA3A partially reverses VEGF effects through binding to neuropilin-1. Stem Cell Res. 2017;22:70–78. 28636974 10.1016/j.scr.2017.05.012

[26] De Vlaeminck Y, Bonelli S, Awad RM, et al. Targeting neuropilin-1 with nanobodies reduces colorectal carcinoma development. Cancers (Basel). 2020;12. 10.3390/cancers12123582 PMC7760077 33266104

[27] Cogan E, Debieve MF, Philipart I. Pepersack T and Abramow M. High plasma levels of atrial natriuretic factor in SIADH. N Engl J Med. 1986;314:1258–1259. 2939346 10.1056/NEJM198605083141921

[28] Liu G, Wang Y, Zhang M, et al. Long non-coding RNA THRIL promotes LPS-induced inflammatory injury by down-regulating microRNA-125b in ATDC5 cells. Int Immunopharmacol. 2019;66:354–361. 30521964 10.1016/j.intimp.2018.11.038

[29] Livak KJ, Schmittgen TD. Analysis of relative gene expression data using real-time quantitative PCR and the 2(-delta delta C(T)) method. Methods. 2001;25(4):402–408. 11846609 10.1006/meth.2001.1262

[30] Pereira D, Ramos E, Branco J. Osteoarthritis. Acta Med Port. 2015;28(1):99–106. 25817486 10.20344/amp.5477

[31] Lin Y, Xing Q, Qin W, et al. Decreased expression of semaphorin3A/neuropilin-1 signaling axis in apical periodontitis. Biomed Res Int. 2017;8724503:2017. 10.1155/2017/8724503 PMC5804370 29457037

[32] Rienks M, Carai P, Bitsch N, et al. Sema3A promotes the resolution of cardiac inflammation after myocardial infarction. Basic Res Cardiol. 2017;112: 42. 10.1007/s00395-017-0630-5 PMC5443852 28540528

[33] Huang B, Zhang Q, Yuan Y, et al. Sema3a inhibits the differentiation of Raw264.7 cells to osteoclasts under 2Gy radiation by reducing inflammation. PLoS One. 2018;13(7):e0200000. 29975739 10.1371/journal.pone.0200000 PMC6033434

[34] Wang Z, Chen J, Zhang W, et al. Axon guidance molecule semaphorin3A is a novel tumor suppressor in head and neck squamous cell carcinoma. Oncotarget. 2016;7(5):6048–6062. 26755661 10.18632/oncotarget.6831 PMC4868739

[35] Teng Y, Yin Z, Li J, et al. Adenovirus-mediated delivery of Sema3A alleviates rheumatoid arthritis in a serum-transfer induced mouse model. Oncotarget. 2017;8(39):66270–66280. 29029510 10.18632/oncotarget.19915 PMC5630410

[36] Sun J, Wei X, Wang Z, et al. Inflammatory milieu cultivated Sema3A signaling promotes chondrocyte apoptosis in knee osteoarthritis. J Cell Biochem. 2018;119(3):2891–2899. 29111592 10.1002/jcb.26470

[37] Yu H, Pei T, Ren J, et al. Semaphorin 3A enhances osteogenesis of MG63 cells through interaction with Schwann cells in vitro. Mol Med Rep. 2018;17:6084–6092. 29484438 10.3892/mmr.2018.8628

[38] Okubo M, Kimura T, Fujita Y, et al. Semaphorin 3A is expressed in human osteoarthritic cartilage and antagonizes vascular endothelial growth factor 165-promoted chondrocyte migration: an implication for chondrocyte cloning. Arthritis Rheum. 2011;63(10):3000–3009. 21953086 10.1002/art.30482

[39] Harper J, Gerstenfeld LC, Klagsbrun M. Neuropilin-1 expression in osteogenic cells: down-regulation during differentiation of osteoblasts into osteocytes. J Cell Biochem. 2001;81(1):82–92. 11180399 10.1002/1097-4644(20010401)81:1<82::aid-jcb1025>3.0.co;2-p

[40] Yang Y, Wang Q, Wang W, et al. Semaphorin 4A antibody alleviates arsenic-induced hepatotoxicity in mice via inhibition of AKT2/NF-kappaB inflammatory signaling. Toxicol Appl Pharmacol. 2021;410:115364. 33290778 10.1016/j.taap.2020.115364

